# Coordinated Metabolic Responses Facilitate Cardiac Growth in Pregnancy and Exercise

**DOI:** 10.1007/s11897-023-00622-0

**Published:** 2023-08-15

**Authors:** Emily B. Schulman-Geltzer, Helen E. Collins, Bradford G. Hill, Kyle L. Fulghum

**Affiliations:** 1 Center for Cardiometabolic Science, Christina Lee Brown Envirome Institute, Department of Medicine, University of Louisville, Louisville, KY, USA; 2 Division of Molecular Medicine, Department of Medicine, University of Minnesota, Minneapolis, MN, USA

**Keywords:** Physiological cardiac growth, Pregnancy, Exercise, Metabolism, Heart

## Abstract

**Purpose of Review:**

Pregnancy and exercise are systemic stressors that promote physiological growth of the heart in response to repetitive volume overload and maintenance of cardiac output. This type of remodeling is distinct from pathological hypertrophy and involves different metabolic mechanisms that facilitate growth; however, it remains unclear how metabolic changes in the heart facilitate growth and if these processes are similar in both pregnancy- and exercise-induced cardiac growth.

**Recent Findings:**

The ability of the heart to metabolize a myriad of substrates balances cardiac demands for energy provision and anabolism. During pregnancy, coordination of hormonal status with cardiac reductions in glucose oxidation appears important for physiological growth. During exercise, a reduction in cardiac glucose oxidation also appears important for physiological growth, which could facilitate shuttling of glucose-derived carbons into biosynthetic pathways for growth. Understanding the metabolic underpinnings of physiological cardiac growth could provide insight to optimize cardiovascular health and prevent deleterious remodeling, such as that which occurs from postpartum cardiomyopathy and heart failure.

**Summary:**

This short review highlights the metabolic mechanisms known to facilitate pregnancy-induced and exercise-induced cardiac growth, both of which require changes in cardiac glucose metabolism for the promotion of growth. In addition, we mention important similarities and differences of physiological cardiac growth in these models as well as discuss current limitations in our understanding of metabolic changes that facilitate growth.

## Introduction

Repetitive or sustained increases in cardiac workload promote hypertrophy of the adult heart, which begins as a compensatory growth process to reduce overall ventricular wall stress and maintain cardiac output. Interestingly, cardiac hypertrophy occurs in response to both pathological (e.g., hypertension) and physiological (e.g., pregnancy, exercise) stressors, with notable distinctions between the cellular and metabolic processes underlying the response to each type of stressor. Although there has been remarkable progress in the understanding of pathological cardiac hypertrophy and the stimuli which lead to harmful remodeling of the heart, much less is known regarding the mechanisms leading to physiological growth of the heart.

Both pregnancy and exercise training increase cardiac workload and drive physiological growth of the heart, which is a reversible phenomenon after parturition or prolonged cessation from exercise, respectively [1, 2]. We understand that these physiological stressors present differently in the heart than pathological stress, but recent studies support that not all physiological growth relies upon the same metabolic mechanisms for growth. By understanding the metabolic mechanisms underlying pregnancy- and exercise-induced cardiac growth, strategies can be developed to optimize cardiovascular health in response to these events and prevent adverse remodeling.

In this review, we address the following questions:

1. What is physiological cardiac growth?
2. What are the metabolic determinants of pregnancy-induced cardiac growth?
3. What are the metabolic determinants of exercise-induced cardiac growth?
4. What causes adverse cardiac events associated with pregnancy and exercise?

## What Is Physiological Cardiac Growth?

Cardiac growth is simply defined as increased mass of the heart, and this typically occurs in response to elevated functional demand. Physiological growth is distinct from pathological hypertrophy in that physiological growth is associated with normal or enhanced cardiac function, increased capillary density, and no induction of the fetal gene program [1, 3]. Additionally, there are distinct metabolic events that distinguish physiological cardiac growth from pathological hypertrophy. One important distinction is that physiological cardiac growth results from intermittent or transient stress and is known to be reversible [2].

The heart is comprised of many cell types. While cardiomyocytes account for about one-third of the cells in the heart, they contribute to at least 70% of cardiac mass [4, 5]. In response to repetitive stimuli, cardiomyocyte growth is facilitated by the addition of sarcomeres, the contractile units of the heart, which form myofibrils and increase the length or width of cardiomyocytes. Parallel addition of sarcomeres increases cardiomyocyte width and leads to concentric cardiac growth—this form of growth is often associated with pressure overload from pathological stimuli such as sustained hypertension or from physiological stimuli such as weightlifting exercises. Serial addition of sarcomeres increases cardiomyocyte length and leads to eccentric cardiac growth—this form of growth is often associated with volume overload from pathological stimuli such as valvular disease or physiological stimuli such as pregnancy or aerobic exercise (reviewed in [6–8]). The focus of this review is on the metabolic mechanisms contributing to eccentric cardiac growth in response to pregnancy and aerobic exercise.

Several molecular events initiate and facilitate cardiac growth. Some studies have shown the requirement of signaling cascades mediated by insulin-like growth factor 1 (IGF-1) [9] and protein kinase B (AKT1) [10], while others imply the necessity of increased expression of genes such as Cbp/P300 interacting transactivator with GluAsp rich carboxy-terminal domain 4 (CITED4) [11] or microRNAs such as miR-222 [12] for physiological cardiac growth. Moreover, recent work highlights the contributions of long noncoding RNAs [13], calcium signaling [14], and lymphangiogenesis [15] in the progression of physiological cardiac growth. There are also mechano-sensing mechanisms and stretch-sensitive ion channels that are thought to coordinate overload of the heart with increased synthesis of proteins [16, 17] and could also be associated with synthesis of membrane components or nucleotides that are important for growing cells. Consistent with the requirement of increased macromolecule synthesis for growth, there are important changes in cardiac metabolism that are unique to physiological cardiac growth and instigated by pregnancy or exercise.

## What Are Metabolic Determinants of Pregnancy-Induced Cardiac Growth?

Physiological cardiac growth occurs during pregnancy as a response to volume overload and is thought to compensate for increased circulatory demands. Many parallels exist between both pregnancy- and exercise-induced cardiac growth, such as both being reversible phenomena that are not associated with perturbations in cardiac function or fibrosis and both involving similar signaling mechanisms such as the activation of the phosphoinosotide 3-kinase (PI3K)/protein kinase B (Akt)/mammalian target of rapamycin (mTOR) pathways [1, 18, 19]. Despite these similarities, pregnancy- and exercise-induced cardiac growth differs in the nature of cardiac overload and the mechanisms that lead to cardiomyocyte growth. These differences have been shown to occur at the transcriptomic level, where late-pregnant hearts had distinct transcriptional profiles compared with exercised hearts [20]. One reason for this difference is that exercise is an acute physiological stimulus, while pregnancy presents a chronic stimulus that is additionally affected by hormonal excursions. Of note, the rises in estrogen and progesterone throughout the course of pregnancy could alter mitochondrial function, as the binding of estrogen to its receptor has been associated with regulation of the metabolic transcription factor, nuclear respiratory factor-1 (NRF-1) (reviewed in [21]), while a recent study reports that progesterone binds a mitochondrial receptor and enhances beta-oxidation in H9C2 cells [22]. The long-term cardiac volume overload during pregnancy is associated with changes not only in hormonal status, but also in both systemic and cardiovascular metabolism.

Systemic metabolism changes dramatically during pregnancy. The early stages of pregnancy are associated with increased anabolic demands in the heart for generating macromolecules and biosynthetic precursors that are necessary to initiate and support maternal cardiac growth; however, in late pregnancy, systemic maternal metabolism shifts more to a catabolic state to divert these constituent building blocks to the fetus to support growth and development [23]. This metabolic switching is necessary to drive systemic adaptations during pregnancy. Glucose is the preferred substrate for fetal metabolism; thus, a reduction in systemic maternal glucose metabolism occurs during pregnancy. This reduction could be a result of the rise in insulin levels during pregnancy and subsequent development of peripheral insulin resistance in the mother [23–25]. The reduction in maternal glucose utilization might spare circulating glucose for fetal growth and also contribute to increased circulating levels of free fatty acids, triglycerides, lactate, and ketone bodies, which together sustain maternal metabolism (maternal systemic metabolism reviewed in [23]). In addition to changes in systemic metabolism, pregnancy is associated with significant cardiovascular adaptations such as increased blood volume, significant changes in hemodynamic parameters (e.g., increased cardiac output and reduced total peripheral resistance), alterations in systolic and diastolic blood pressures, and the development of reversible cardiac growth [26]. Even with this knowledge, little is known about the underlying metabolic mechanisms contributing to pregnancy-associated cardiac growth, likely due to the lack of inclusion of pregnant women and animals in clinical and preclinical studies, as well as the reduced awareness of pregnancy-associated cardiovascular adaptations and disease conditions.

**Fig. 1:**
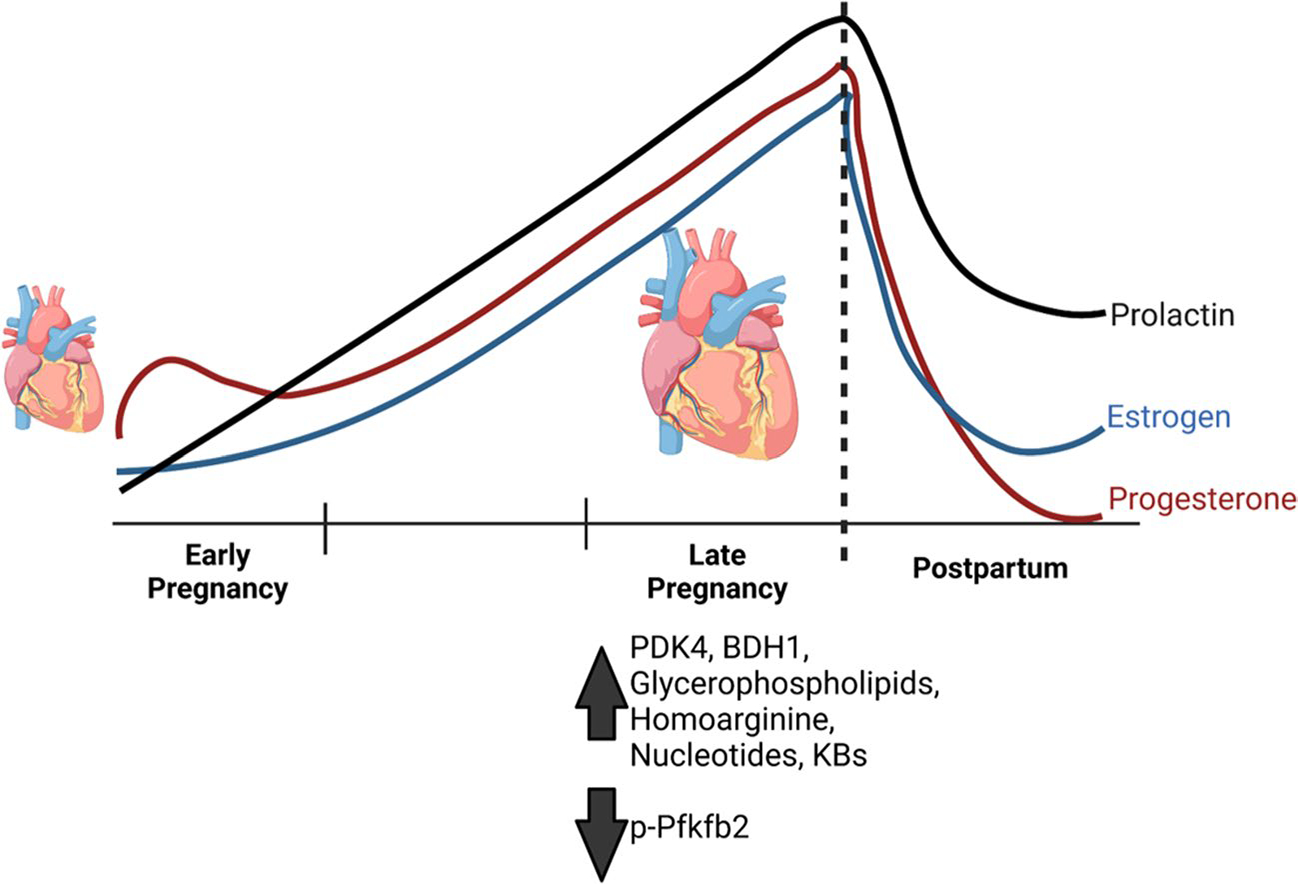
Coordinated metabolic responses facilitate pregnancy-induced cardiac growth. Pregnancy-induced changes in circulating metabolite abundances lead to significant changes in the heart during late pregnancy when cardiac growth occurs. Increased Pdk4 and Bdh1 expression and reduced Pfkfb2 activity could facilitate the metabolic flexibility needed to promote anabolism in the heart and could be mediated by the rising estrogen, progesterone, and prolactin levels. Hormone levels represented in the figure follow trends from human data. Figure made using “http://biorender.com”

Despite the associations between changes in cardiac metabolism and ventricular remodeling, little is known about the extent of the metabolic changes in the maternal heart. Recent key metabolic findings in the maternal heart are shown in Fig. 1. Evidence suggests that reductions in cardiac glucose catabolism are essential for cardiac growth during pregnancy. Studies performed in the 1990s showed that changes in pyruvate oxidation occurred in the maternal heart [27], and, more recently, Liu et al. showed a progesterone-dependent increase in the expression of pyruvate dehydrogenase kinase 4 (Pdk4) in the late-pregnant heart [28]. In addition, those authors showed that inhibition of Pdk4 with dichloroacetate during late pregnancy inhibits cardiac growth, indicating that a reduction in cardiac glucose catabolism is required for pregnancy-induced cardiac growth. Recent findings corroborate these results, where Fulghum et al. showed a significant increase in Pdk4 expression and concomitant reduction in the phosphorylation of phosphofructokinase during late pregnancy [29••], similar to what has been observed in the heart following acute bouts of exercise [30]. In addition, increases in circulating ketone bodies and cardiac expression of Bdh1 were elevated in the maternal heart, seeming to follow the arc of pregnancy-induced cardiac growth [29••]. This is intriguing because ketone bodies are typically associated with metabolic remodeling during cardiac pathology [31, 32] but might play an important role in metabolic remodeling observed in the maternal heart during pregnancy. However, the extent to which ketone bodies influence growth of the maternal heart remains unknown. In addition, during pregnancy, the heart is thought to consume more lactate than glucose; however, the contribution of lactate to maternal cardiovascular adaptations has been vastly understudied. Therefore, it is necessary that future studies assess the contributions of these circulating metabolites to maternal systemic and cardiovascular adaptations.

Increased glucose flux into ancillary biosynthetic pathways of glucose metabolism has been shown to support anabolic growth, but few studies have interrogated the full extent of metabolomic changes in the maternal heart. Recently, it was demonstrated that metabolites associated with these anabolic, pro-growth pathways, such as nucleotides, glycerophospholipids, and amino acids, were increased in the late pregnant murine heart and in 1-week post-birth murine hearts, which coincided with pregnancy-induced cardiac growth [29••]. Similar metabolite changes have been observed in newborn ovine hearts, suggesting a degree of metabolic coordination between the maternal-fetal unit. Of the amino acid metabolites identified, the urea cycle and polyamine metabolites were the most abundant in the hearts of late pregnant and post-birth mice. This included the urea cycle-associated metabolite, homoarginine, which dramatically increased during late pregnancy, similar to changes observed in the acute exercised female heart [33•], and is consistent with documented changes in nitric oxide during pregnancy [34]. This is of interest because the importance of the urea cycle has yet to be thoroughly interrogated in the heart. In addition, polyamines have been associated with cardiac hypertrophy, but few studies associate increased polyamines with cardiac growth during pregnancy. Therefore, the extent to which the urea cycle and polyamine metabolites contribute to the physiological and metabolic remodeling of the maternal heart currently warrants additional investigation. Despite this knowledge, studies have only shown a snapshot of static metabolite changes rather than information on specific metabolite flux, which would shed more light on the metabolic changes occurring in the heart during pregnancy.

## What Are Metabolic Determinants of Exercise-Induced Cardiac Growth?

**Fig. 2:**
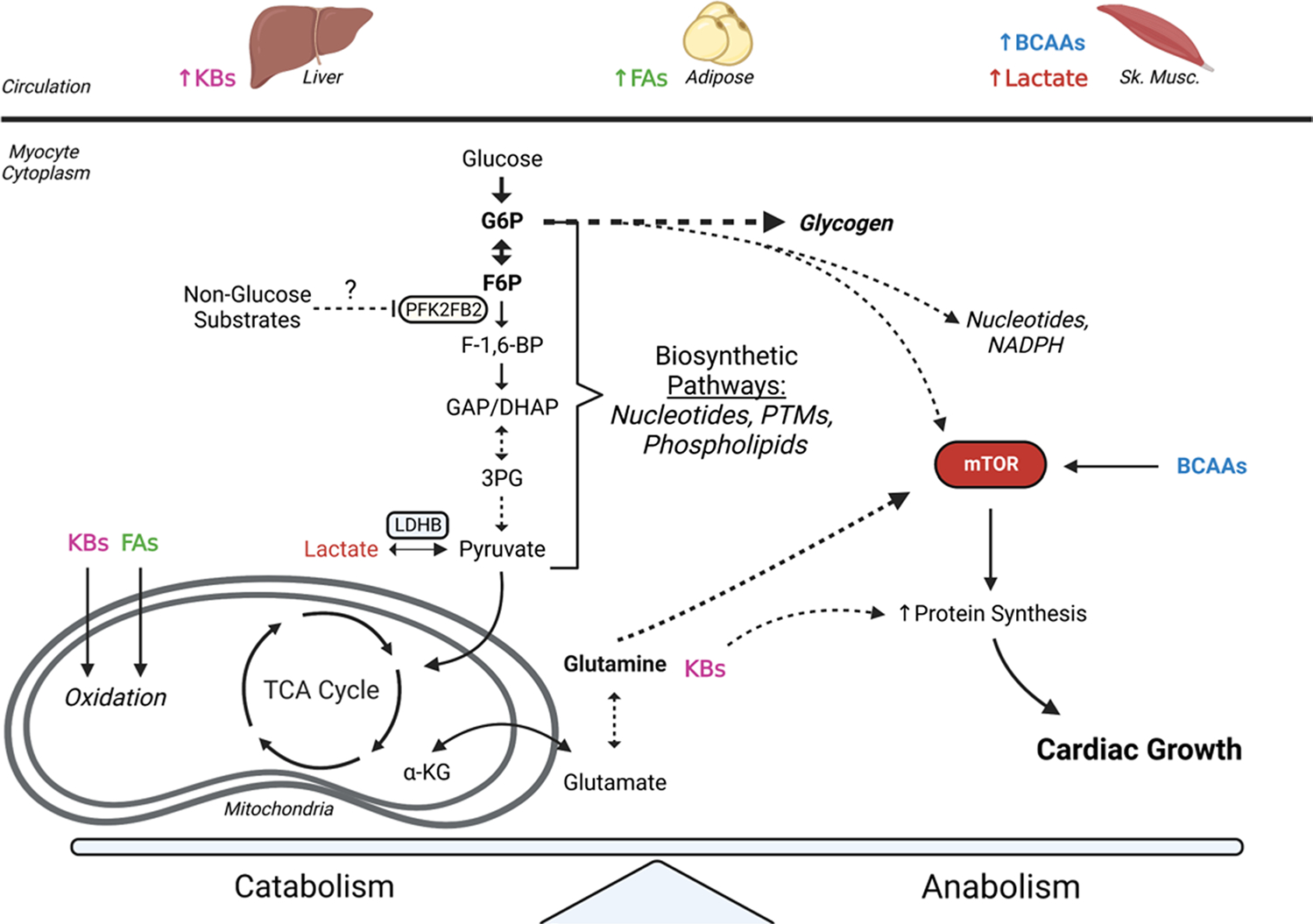
Coordinated metabolic responses facilitate exercise-induced cardiac growth. Exercise-induced elevations in circulating metabolites are taken up by the heart and utilized for both maintenance of contractile function as well as biosynthetic purposes. Reductions in cardiac PFK2FB2 activity influences glucose metabolism and may spare glucose-derived carbons for incorporation into biosynthetic pathways for promotion of cardiac growth. Elevations in fatty acids (FAs), branched-chain amino acids (BCAAs), glutamine, and ketone bodies (KBs) may work together to facilitate cardiac growth from routine exercise. Figure made using “http://biorender.com”

Regular exercise training is associated with numerous health benefits, including reductions in the risk for heart disease and adverse cardiovascular events [35]. Acute responses to exercise involve transient molecular and metabolic changes that facilitate augmented cardiac output and excitation-contraction coupling, while chronic responses to exercise involve sustained changes and structural adaptations that, like pregnancy-induced cardiac growth, reduce wall stress incurred during periods of elevated cardiac output [36]. While several molecular events are well-known to enable physiological growth of the heart, the metabolic underpinnings of physiological cardiac growth remain less evident. Current understanding of metabolic contributions to exercise-induced cardiac growth is highlighted in Fig. 2.

During exercise, higher rates of catecholamine-induced adipose tissue lipolysis and skeletal muscle contraction increase the concentration of fatty acids and lactate in circulation up to 2.4 mM [37] and nearly 10 mM [38, 39], respectively. As such, the heart increases its energetic reliance on these substrates while reducing the contribution of glucose oxidation to ATP production [7]. While at rest, fatty acids contribute up to 70% of oxidative metabolism in the heart [40, 41]. The contribution of fatty acids to energy production remains high during most bouts of exercise; however, during intense exercise, lactate may contribute up to 90% of total oxidative metabolism in the heart because of its high concentration in circulation [42]. Interestingly, it appears that fat oxidation and triacylglycerol turnover may increase in the presence of lactate [43, 44], which could implicate synergistic roles of lactate and fatty acids in the heart during exercise. Moreover, chronic exercise leads to elevations in cardiac expression of genes involved in fatty acid catabolism and transport [45], which increases cardiac fatty acid oxidation in the exercise-trained heart [46]. While acute exercise increases circulating lactate abundance and utilization by the heart, prolonged training appears to have minimal effects on lactate oxidation capacity or the abundance of mitochondria-associated lactate dehydrogenase [47].

During exercise, the increases in fatty acid and lactate oxidation in the heart are paired with a reduction in glucose oxidation [7]. This transient decrease in glucose utilization could be due to the heightened availability of competing substrates and may be critical for the adaptive response of the heart to exercise. During exercise, there appears to be a transient reduction in cardiac activity of 6-phosphofructo-2-kinase/fructose-2,6-bisphosphatase (PFKFB2) activity; however, following exercise, cardiac PFKFB2 activity appears to increase. Expression of a kinase-deficient PFKFB transgene in the heart resulted in constitutive reduction in cardiac glycolysis and a physiological cardiac growth phenotype similar to that of an exercise-trained heart [30], suggesting that changes in cardiac glucose metabolism are sufficient to elicit physiological growth. The increase in cardiac glucose oxidation following exercise could be facilitated by increased translocation of GLUT4 to the cardiomyocyte membrane [48], and since less than 50% of glucose that enters the heart is fated for complete oxidation [49], increased glucose uptake may serve the purpose of shuttling glucose-sourced carbons into biosynthetic pathways. Interestingly, an increase in cardiac glycogen accumulation was observed immediately following exercise and simultaneously with reductions in glucose oxidation [30], which could facilitate a cycle of glucose storage and subsequent utilization [50] in the heart following exercise.

Ketone bodies, branched-chain amino acids (BCAAs), and glutamine contribute much less to cardiac energetics under normal conditions (<15% overall) than lactate, fatty acids, and glucose [7]. The ketone bodies β-hydroxybutyrate and acetoacetate have been shown to be important in heart failure by maintaining metabolic capacity in working heart models [32]. Although the failing heart relies upon ketone bodies, high circulating levels of ketone bodies in the absence of other substrates are associated with functional impairment of the heart [51, 52]. However, even in the presence of other substrates, ketone bodies have been shown to inhibit both glucose [53–55] and fatty acid oxidation [56, 57] in the heart. It is unknown if the heart uses ketone bodies solely for energy provision, as previous studies suggest ketones are anticatabolic in nature [58], and elevations in β-hydroxybutyrate reduce leucine oxidation and concomitantly increase skeletal muscle protein synthesis [59], which is possibly mediated by mechanisms that enhance mTOR signaling. Furthermore, β-hydroxybutyrate has been shown to inhibit histone deacetylases (HDACs) [60], which could activate transcription of genes that regulate glucose metabolism and cardiac adaptation to exercise. Additionally, metabolic pathway activity is influenced by the binding of β-hydroxybutyrate to hydroxycarboxylic acid receptor 2 (HCAR2) and free fatty acid receptor 3 (FFAR3). Binding of β-hydroxybutyrate to these G-protein coupled receptors has been associated with reductions in adipose lipolysis [61, 62], which further suggests that β-hydroxybutyrate could be an anti-catabolic metabolite and may implicate a temporal role in its major functions following exercise. It remains unknown, however, how these effects carry over to cardiac muscle following exercise.

One study showed that an acute bout of exercise increases circulating and cardiac abundance of β-hydroxybutyrate to a greater extent in female FVB/NJ mice than males [33•], which could indicate a sex-dependent response to cardiac utilization of ketone bodies. Other studies showed that ketogenic diets increase exercise performance in male, but not female rodents (reviewed in [63]). These findings could implicate a role of ketone body metabolism in exercise-induced cardiac growth since studies suggest that female C57BL6/J mice have greater cardiac growth responses to exercise training than males [64]; however, in humans, it appears that males have greater cardiac growth responses to exercise training than females [65]. Nevertheless, the role of cardiac ketone body metabolism in exercise-induced cardiac growth is unknown.

Branched-chain amino acids contribute minimally to myocardial oxygen consumption (<5%) but are important for protein synthesis and muscle growth [66]. In fact, a recent study indicates that myocardial accumulation of BCAAs is required for cardiomyocyte hypertrophy and is mediated in part by the coordination of glucose and BCAA utilization in the heart through Kruppel-like factor 15 (Klf15) to promote hypertrophic signaling [67•]. While circulating levels of BCAAs are dependent on exercise intensity [68, 69], myocardial abundance of BCAAs have been shown to increase following a bout of exercise but return to normal levels within 24 h [33•]. Interestingly, provision of a diet high in BCAAs for just 4 h at the end of the active phase leads to significant cardiac growth and hypertrophy of cardiomyocytes in mice [70•], which indicates that increases in cardiac BCAA abundance could be an important regulator of cardiac growth. That one high BCAA diet at the end of the active phase promotes cardiac growth implies metabolic responses may be dependent upon the time of day. Corroborating this notion is the finding that time of day significantly influences cardiac metabolite abundances following one bout of exercise. Of note, there were more changes in cardiac metabolite abundances following exercise in the early active phase (i.e., morning) compared with early rest phase (i.e., evening) exercise; however, early rest phase exercise increased cardiac abundance of corticosterone and 5-aminoimidazole-4-carboxyamide ribonucleotide (AICAR) [71•], the latter of which regulates the metabolic controller, AMP kinase (AMPK) [72]. The coordination of BCAA metabolism with other substrates in the heart may enhance hypertrophic signaling and spare glucose-sourced carbons for biosynthetic purposes; however, this hypothesis needs further examination.

While the contribution of glutamine to cardiac energetics is relatively low [7], its importance in anaplerosis and activation of biosynthetic pathways [73] might highlight its requirement for growth responses in the heart. In particular, glutamine has been shown to activate mTOR [74] and has been associated with supplying the Krebs cycle with metabolites via its interconversion to the amino acid glutamate [66] and then to α-ketoglutarate [75]. Furthermore, one group recently showed that L-glutamine supplementation reduces cardiac tissue injury following exercise [76], which may suggest an important, coordinated role of glutamine in tissue repair responses.

Taken together, exercise presents a transient stress response to the heart for maintenance of elevated cardiac output. While the heart responds via efficient oxidation of many substrates, it remains unclear how exercise influences substrate utilization to promote anabolism in the heart. Additionally, as metabolite abundances in circulation and in the heart change over time following exercise, it is important to consider temporal effects of cardiac substrate utilization during and following exercise to fully understand how exercise-induced changes in cardiac metabolism promote growth of the heart.

## What Causes Adverse Cardiac Events Associated with Pregnancy and Exercise?

Although awareness of pregnancy-associated cardiovascular complications has increased in recent years, little advancement has been made in elucidating the molecular and metabolic mechanisms underpinning these diseases. This is likely due, in part, to a consequence of there being little information available regarding how these mechanisms change during normal pregnancies or, more broadly, in the female heart at all. Metabolic perturbations are seen in most non-obstetric cases of heart failure, so it seems reasonable to hypothesize that these also occur in the setting of pregnancy-associated cardiovascular complications. In support of this, it was recently shown that human iPSC-derived cardiomyocytes from patients with peripartum cardiomyopathy (PPCM) have altered lipid metabolism [77] compared with healthy hearts, while another study showed that mTOR dysregulation in the heart precipitates PPCM-like cardiac changes [78]. In addition, many preclinical models of pregnancy-associated cardiovascular diseases, such as pre-eclampsia and PPCM models, exhibit metabolic dysfunction. For example, cardiac-specific knockout of signal transducer and activator of transcription 3 (STAT3) or cardiac-specific knockout of peroxisome proliferator-activated receptor-gamma coactivator (PGC-1α) in mice not only results in a PPCM-like phenotype [79, 80] but also results in major metabolic changes in the heart. However, at this point, no studies are available that characterize metabolic flux and substrate flexibility in the maternal heart under these disease conditions. Nonetheless, many circulating biomarkers identified in pre-eclampsia patients, such as glutamate [81], tryptophan metabolites [82], and arachidonic acid metabolites [83], likely have a significant impact on metabolism and should be interrogated further. Despite these insights, the extent to which changes in cardiac metabolism play a role in pregnancy-associated diseases remains unclear.

Even though the cardiovascular complications of pregnancy might be much more prevalent than those associated with exercise, there are still important considerations for heart health when following an exercise training regime. While regular exercise has numerous benefits, both excessively prolonged and intense exercise may actually increase acute cardiac events and pathological remodeling of the heart (reviewed in [84, 85]). These types of exercise (i.e., ultramarathons) often lead to elevations in circulating cardiac troponins, with the intensity of exercise positively associated with troponin levels [86]. Furthermore, intense exercise can lead to 10-fold elevation in B-type natriuretic peptide, a marker of cardiac injury, with long-term strenuous exercise being additionally associated with myocardial fibrosis and even calcification of coronary arteries [84]. Although a single bout of strenuous exercise may depress cardiac function and mildly resemble cardiac injury [87, 88], the changes in cardiac function usually recover within a couple days following exercise [89]. Taken together, there appears to be a threshold of exercise duration and concomitant recovery that define physiological and pathological remodeling of the heart following exercise. While short-lived elevations in injury markers are observed to much lesser extent in typical exercise exertions, it remains unknown if and how these contribute to cardiac adaptation to exercise.

## Conclusions

Physiological cardiac growth enables the heart to maintain elevated output during pregnancy or exercise with reduced wall stress. Coordination of myocardial substrate utilization balances substrate oxidation for provision of ATP with anabolic demands of growing cells. The mechanisms facilitating this type of growth are different than those responding to pathological stimuli; even more, the mechanisms by which pregnancy- and exercise-induced cardiac growth occur are also somewhat different. For example, both types of physiological cardiac growth appear to require reductions in glucose oxidation, which could divert glucose-sourced carbons into biosynthetic pathways, but this phenomenon might be regulated by Pdk4 in pregnancy-induced growth [28], whereas it might be regulated by PFKFB2 in exercise-induced growth [30]. Furthermore, while exercise is associated with changes in metabolite abundances in the heart [33•], the late-pregnant heart and postpartum heart are seemingly associated with a greater number of significantly changed metabolites that facilitate growth and maintenance of function [29••]. Interestingly, metabolite abundances in the female mouse heart following exercise change more than in the male heart, yet it remains unknown how the metabolic landscape of a pregnant heart might compare with the exercise-trained female heart. While several similarities seem to exist regarding physiological growth of the female heart, such as elevations in cardiac abundance of homoarginine and in circulating ketone bodies, the precise metabolic mechanisms facilitating cardiac growth in response to pregnancy or exercise remain unresolved. Since homoarginine and ketone bodies are likely liver-derived, it is important that future studies on physiological cardiac growth examine the liver-heart axis when assessing metabolism.

Currently, there are limitations to ascertaining mechanisms by which the heart grows in response to physiological stimuli or mechanisms by which the adapted heart reverts to normal size. Murine models of pregnancy are routinely used, but to date, none have examined the contribution of myocardial substrates to metabolite pools through isotope tracing, which would allow for flux analyses and would illuminate relationships between biosynthetic pathways. Similarly, in exercise, several studies perform untargeted metabolomics in the heart or use isotope tracers for short durations, which might not be adequate for deep network tracing of substrate contributions into major biosynthetic pathways. Additionally, studies on exercise-induced cardiac growth typically focus on aerobic exercise, with few studies examining the effects of resistance exercise on cardiac growth. One important reason may be the lack of consensus on murine models for weightlifting, as weightlifting maneuvers of humans are difficult to simulate in quadrupedal murine species; however, a recent study suggests there is a model that elicits similar skeletal muscle responses in mice as weightlifting does in humans [90], which could be a step forward in assessing metabolic determinants of resistance exercise-induced cardiac growth.

Most of our knowledge regarding physiological cardiac growth comes from the contribution of cardiomyocytes or whole-heart studies, but recent work (reviewed in [91]) highlights the important roles of metabolism and cell signaling in non-cardiomyocytes in the development of cardiac growth. We understand that a coordinated metabolic response facilitates growth of the heart, but future work is needed to integrate findings and to additionally elucidate the contribution of non-cardiomyocytes to cardiac growth.
